# Standard Gum Elastic Bougie Versus Flexible Tip Bougie: Evaluation of Airway Adjuncts for Intubation by Paramedics in Entrapped Manikins with Difficult Airway Access—A Randomised, Controlled Trial

**DOI:** 10.3390/healthcare12222232

**Published:** 2024-11-08

**Authors:** Dawid Aleksandrowicz, Paweł Ratajczyk

**Affiliations:** 1 Department of Anaesthetics, Intensive Care Medicine and Pain Therapy, Mazovian Specialist Hospital, 26-600 Radom, Poland; 2 Department of Anesthesiology and Intensive Therapy, Medical University of Lodz, 90-153 Lodz, Poland; pawel.ratajczyk@umed.lodz.pl

**Keywords:** tracheal intubation, trauma, entrapped patients, introducer, flexible tip bougie

## Abstract

**Introduction:** Airway management in pre-hospital settings is often challenging and difficult to perform. This is particularly true during tracheal intubation of entrapped patients with difficult airway access. There are various airway adjuncts available in the current practice. Their aim is to facilitate tracheal intubation. One of the recently introduced devices is the flexible tip bougie. The aim of this study was to evaluate the flexible tip bougie for intubation in a simulated condition of an entrapped trauma patient with simultaneous cervical spine immobilisation. **Methods:** An intubation manikin with the cervical collar on was placed on and secured to the driver’s seat of a passenger car. The car was then positioned on its left side. The first attempt success rate as well as the intubation and successful ventilation time were recorded. The ease of use by the operator and user-friendliness were also assessed. **Results:** The standard gum elastic bougie required the shortest mean intubation to successful ventilation time, 38.4 s (±9.6) vs. 41.4 s (±6.8), *p* = 0.46. The first attempt success rate was higher in the standard gum elastic bougie group, 92% vs. 88%, *p* = 0.04. There were no failed intubations when the standard gum elastic bougie was used. The flexible tip bougie was found to be more difficult to use and less user-friendly, 7.6 (±1.5) vs. 5.8 (±2.9), *p* = 0.02. **Conclusions:** The standard gum elastic bougie was superior in terms of the first attempt success and the time required for intubation and successful ventilation. Moreover, it was found to be easier to use and more user-friendly.

## 1. Introduction

Trauma care forms a challenge to modern-day healthcare systems worldwide. The number of trauma patients worldwide is increasing. Furthermore, trauma is the third leading cause of death among those aged 1–44 years [1]. It is worth noting that an important contributor to these deaths is road traffic collisions (RTCs) [2].

According to the World Health Organization (WHO), RTCs represent the leading mechanism of injury in major trauma as well as the leading cause of deaths among those aged 5–29 years worldwide [3]. RTCs are not a uniform entity. They can be divided into different types, which include the following: lateral impact, frontal collision, near-side collision, and roll over. Each of them carries the potential risk that victims may not be able to leave the vehicle unassisted. Such victims are considered entrapped. They are at higher risk of sustaining serious injuries compared to those who can leave the vehicle unassisted [4]. This is of particular importance in relation to airway compromise and inadequate ventilation. The management of such patients is often more challenging and difficult due to the restricted access as a result of the final position of the vehicle after a crash. Therefore, the entire process should ideally be performed in a timely manner by a skilled practitioner.

Airway management is a crucial element of the pathway of care of the injured. The current practice follows the well-known and widely used AcBCDE algorithm. Airway management plays an important role and is performed simultaneously together with cervical spine immobilisation or MILS (Manual In-line Stabilisation) [5]. This in turn can worsen the glottic view and hence make tracheal intubation difficult [6,7,8,9]. Of the available techniques, tracheal intubation is still regarded as the gold standard when it comes to trauma patients.

The main aim of airway management in the injured is the maintenance of airway patency with adequate ventilation and oxygenation. This can be achieved with prompt intubation. Tracheal intubation, especially in pre-hospital settings, is often difficult and may be associated with various complications [10,11,12,13]. Therefore, this procedure should be performed by a skilled and experienced practitioner. In the current practice, there are various tools available, which may be of help during intubation. One of such adjuncts is an airway introducer and the most often used is the standard gum elastic bougie. Its main purpose is to facilitate tracheal intubation. These devices may become very useful in difficult airway situations, both in out-of-hospital and in-hospital settings [14,15,16].

In recent decades, there has been a considerable development of various new airway adjuncts available in clinical practice. These devices are predominantly manufactured so that tracheal intubation is easier. Most of the recently developed tracheal tube introducers are modifications of the standard gum elastic bougie. An example of such a device is the STIG (Steerable Tracheal Intubation Guide), also known as the flexible tip bougie (Construct Medical Pty Ltd., Hawthorn, Australia). The STIG has some unique features, which include a flexible and steerable tip with bright phosphorus coating and slider tabs. The former makes it easier to navigate difficult airways and improves the visibility of the tip, especially with the ultra-violet light, which may be used by some laryngoscopes. The latter enables to flex the tip of the introducer.

The aim of this study was to evaluate the new flexible tip bougie for intubation in a simulated scenario of an entrapped trauma patient with simultaneous cervical spine immobilisation. The new introducer was evaluated by experienced paramedics in a situation where access to the RTC victim was difficult. The performance of the flexible tip bougie was compared against the standard gum elastic bougie (SGEB).

## 2. Methods

This study has been approved by the University of Radom’s Ethics Committee (KB/10/2023; Head: Prof. Z. Stojcev; Date: 11 October 2023).

Written informed consent was obtained from all study participants. Only fully qualified paramedics participated in the study. Their experience varied between four and eight years of practice after completion of paramedic training. None of the study participants had used the STIG previously, but they all had a routine intubation experience with the standard gum elastic bougie (having performed more than 40 intubations).

**Figure 1:**
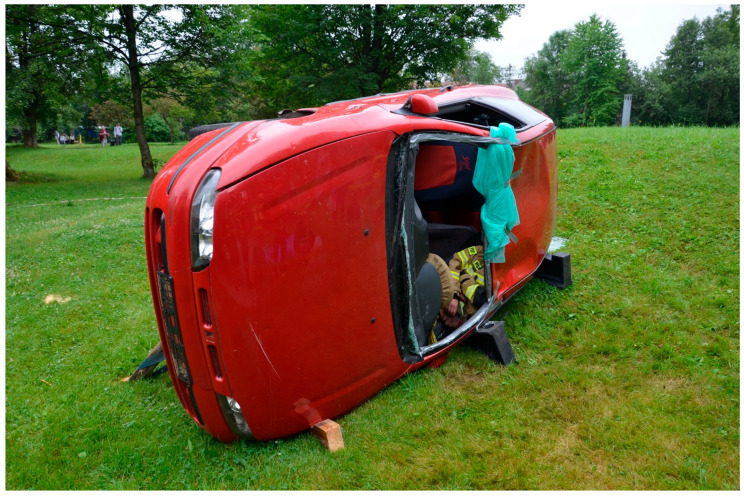
The final position of the passenger car used for the study.

**Figure 2:**
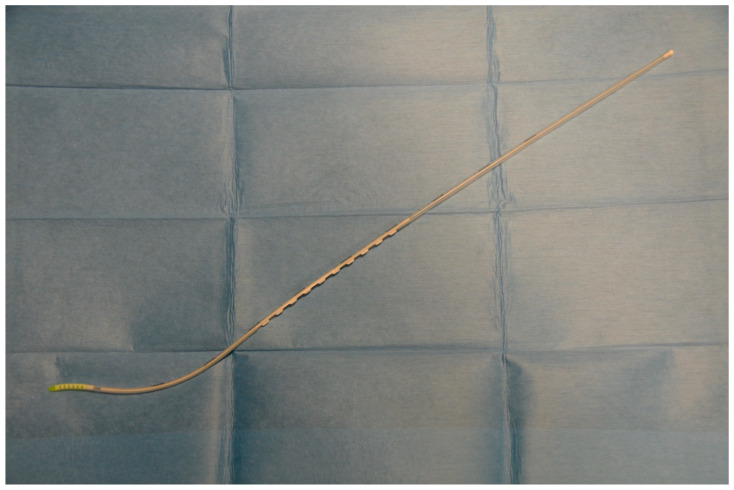
The flexible tip bougie.

Before the start of the study, all paramedics took part in a 10 min lecture, during which it was explained how the STIG should be used. Following this presentation, all participants could familiarise themselves with the new equipment and practice for 30 min. A skill station was set up for the practice. It comprised an AT Kelly Torso intubation manikin (Laerdal Medical AS, Stavanger, Norway). A Patriot^®^ cervical collar (Össur hf., Reykjavik, Iceland) was applied in order to achieve a reduced movement of the cervical spine. After completion of the 30 min training, the intubation manikin with the cervical collar on was placed on the driver’s seat of a compact passenger car (FIAT S.p.A., Turin, Italy). The car was then turned on its left side and secured in place by firemen from a local fire brigade (Figure 1). An opening was created after removal of the windscreen and access to the manikin was only allowed through it. There was no unified technique of intubation, as each of the paramedics performed it based on their experience. However, all paramedics intubated the manikin facing it in a kneeling position. Such technique required the standard laryngoscope to be held like a pickaxe hence the term ‘pickaxe approach’. Such technique was the most often preferred by the participants. The pickaxe approach required the laryngoscope to be held in the dominant hand and the introducer in the non-dominant hand. Such an approach is different to the routine intubation technique and has been described in detail elsewhere [17]. A number was allocated to each of the studied devices, i.e., 1 for the STIG (Figure 2) and 2 for the standard gum elastic bougie (SUMI Sp. z o.o., Sp. K., Sulejówek, Poland). Each paramedic was asked to randomly give a number (either 1 or 2) and was then given the corresponding introducer to use. The maximum number of intubation attempts was limited to three per device. The time required to intubate and successfully ventilate (T_i_) the manikin was recorded. It was measured using a stopwatch of a mobile phone (Apple Inc., Cupertino, CA, USA). The T_i_ was measured from picking up the studied device by a paramedic until successful ventilation was confirmed. Efficacy of intubation and the ease of use by the operator were also assessed. An 11 point numerical rating scale (NRS) was utilised for the latter. An NRS score of 0 corresponded to a very difficult-to-use device, while the score of 10 indicated an easy-to-use tracheal tube introducer. All intubations were performed with a size 7.5 tracheal tube (SUMI Sp. z o.o., Sp. K., Sulejówek, Poland). A manual resuscitator (Ambu A/S, Ballerup, Denmark) was used for ventilation and was readily available to the paramedics. A standard size 3 Macintosh-blade laryngoscope (New Waseem Trading Co., Sialkot, Pakistan) was used for direct laryngoscopy. The study participants performed all intubations with each of the two tracheal tube introducers. A failed intubation was defined as an attempt that lasted longer than 120 s or an attempt during which the trachea could not be intubated. Only paramedics who failed to intubate were allowed another attempt. All the obtained data were analysed using Microsoft Office Excel 2021 spreadsheet (Microsoft Corporation, Redmond, WA, USA) and GraphPad Prism (GraphPad Software, Boston, MA, USA, https://www.graphpad.com/features). The Kolmogorov–Smirnov test was used to determine whether the analysed variables matched the characteristics of a normal distribution. A paired Student *t*-test and the Wilcoxon signed-rank test were used for data analysis. Based on our previous, unpublished, pilot study, we assumed that the overall success rate of intubation would be 90% (α = 0.05, 2-sided, β = 0.1) and that 46 participants were required. The final adjusted sample size, allowing a drop-out rate of about 10%, was 50, and this was the final number of participants enrolled in the study. A *p* value of less than 0.05 (*p* < 0.05) was considered statistically significant.

## 3. Results

Fifty paramedics with a mean of 6.4 years of active pre-hospital work participated in the study. The majority of participants were male (*n* = 33), compared to female participants (*n* = 17).

The shortest time to intubation and successful ventilation was achieved when the standard gum elastic bougie was used, 38.4 s (±9.6) vs. 41.4 s (±6.8), *p* = 0.46 (Table 1). 

There were three failed intubations in the flexible tip bougie group. Two of them were attributed to the length of the procedure breaching the allowed 120 s mark. There was also one oesophageal intubation when the STIG was used. No failed intubations were reported in the standard gum elastic bougie group. However, four intubations were successful at the second attempt out of the three allowed (Table 2).

## 4. Discussion

Airway management in pre-hospital settings, particularly in trauma patients, is often difficult and challenging. Victims who are unable to leave the vehicle unassisted form a specific group of patients because there is often a difficult or limited access to them [18,19]. Therefore, one has to bear in mind that airway management and the entire resuscitation process may be extremely difficult in this patient population.

The number of motor vehicles worldwide is increasing. Therefore, there is not only a greater risk of road traffic collisions but also a potential for RTCs victims to be trapped in their vehicles. That is why it is important, from a practical point of view, to know which airway equipment performs better and which ones to use in such challenging settings. In the authors’ opinion, this is the reason for all new airway devices to be carefully evaluated and studied in different trauma-related scenarios. Pre-hospital patients’ resuscitation and airway management, in particular, are key in improving patients’ outcome and reducing mortality. These actions should be performed in a timely manner by experienced personnel with adequate skills, knowledge and equipment.

The aim of this study was to evaluate a recently introduced STIG device. The flexible tip bougie was evaluated by fully qualified paramedics in a situation with difficult access to a road traffic collision victim. To the authors’ knowledge, this is the first study to compare this introducer in a simulated scenario involving an entrapped RTC victim.

The evidence of the flexible tip bougie performance in trauma patients is sparse in the current literature.

The primary outcome measures of our study were the time required for intubation and successful ventilation and the first attempt success rate.

In a recent study by Mahli et al. the flexible tip bougie was evaluated in simulated difficult airway conditions [20]. The authors compared the performance of both the flexible tip bougie and the SGEB. They found the former to be superior with regards to the first pass success rate, 98.4% vs. 85.5%, *p* = 0.008, and the median intubation time, 32 s vs. 41.5 s, *p* < 0.001. These findings were contradictory to the results of our study, as both of the intubation time and the first attempt success rate were in favour of the standard gum elastic bougie. Oxenham et al. demonstrated that both the SGEB and the flexible tip bougie were comparable, with no significant differences in the first-attempt success rate or the total intubation time [21]. However, neither of the bougies was evaluated in a difficult airway scenario and both were used to aid in a video laryngoscopic intubation.

Frass et al. [22] evaluated both the SGEB and the STIG used by nurses in a difficult airway setting. They found the flexible tip bougie to be more efficient in terms of the first-pass success rate. Furthermore, the use of the STIG was associated with shorter intubation times.

In a recent study that assessed both the STIG and the standard gum elastic bougie during cardiopulmonary resuscitation in an adult manikin, the authors concluded that the flexible tip bougie was superior both in terms of the first attempt success rate as well as the mean intubation time [23]. Both tracheal tube introducers were evaluated by paramedics. Rützler et al. [24] evaluated the flexible tip bougie in six different scenarios of which one included cervical spine immobilisation. In their study, the flexible tip bougie outperformed the standard gum elastic bougie in terms of both the time to intubation and the first attempt success rate, 37 s vs. 46 s, *p* < 0.001 and 94% vs. 81%, *p* > 0.05, respectively. Of note is the fact that both of the studied devices were assessed by experienced anaesthetists.

One of the secondary outcome measures of our study was the efficacy of intubation. We found the standard gum elastic bougie to be more efficient as there were no failed intubations when this introducer was used. No failed intubations with either of the two evaluated devices were reported by other investigators [21,22,24], even though both of the studied tracheal tube introducers had to be used during the second or third attempt to achieve successful intubation.

The ease of use and user-friendliness were also assessed in our study. All study participants were asked to rate both of the evaluated devices using the eleven-point NRS. The score ‘10’ represented the most easy-to-use and user-friendly equipment. The standard gum elastic bougie was found to be superior to the flexible tip bougie in our study. Similar results were reported by Rützler et al. in a simulated scenario with cervical spine immobilisation [24]. Contrary to our results, some authors have found that the flexible tip bougie was easier to use and more user-friendly when compared to the standard gum elastic bougie [21,25].

Several limitations of this study exist. One of them is a relatively small sample size that consisted of fifty paramedics. Another is the fact that this was a manikin study. Certain anatomical differences exist between commercially available manikins and human individuals. These are not solely related to the materials used (often less compliant than human soft tissues) but also to the lack of secretions and different dimensions in the airway space between the epiglottis and the posterior pharyngeal wall [26]. It may be difficult to extrapolate the findings of such a study to the human population. This has to be kept in mind when considering the results of this study. However, one has to bear in mind that manikin studies are both justifiable and ethical [27,28]. Furthermore, manikin studies are a recognised tool in the early stages of a new device assessment process before such a device enters the market. Other limitations include the lack of double-blinding and the inclusion of experienced paramedics who used the SGEB previously, which may be a potential source of selection bias.

## 5. Conclusions

This study has found that the standard gum elastic bougie was superior in terms of the time to intubation and successful ventilation and the first attempt success rate. Moreover, the standard gum elastic bougie was found to be easier to use and more user-friendly. The flexible tip bougie was associated with an increased risk of failed intubation. The older device is not inferior to the newer one when used for intubation with simultaneous cervical spine immobilisation in an entrapped trauma patient with restricted access.

## Figures and Tables

**Table 1:** Comparison of Studied Devices (T_i_).

Studied Device	Insertion to Intubation and Successful Ventilation Time (Ti) [s]			
	Min	Max	Mean (SD)	*p* Value
Standard Gum Elastic Bougie	23.5	52.7	38.4 (9.6)	0.46
Flexible tip Bougie	25.2	81.3	41.4 (6.8)	

SD—Standard Deviation, s—seconds.

**Table 2:** Efficacy of Intubation.

Number of Attempts	Studied Device				*p* Value
	Standard Gum Elastic Bougie		Flexible Tip Bougie		
	N	%	N	%	
1	46	92	44	88	0.04
2	50	100	45	90	
3	-	-	47	94	

The flexible tip bougie was found to be more difficult to use and less user-friendly when compared to the SGEB 7.6 (±1.5) vs. 5.8 (±2.9), *p* = 0.02 (Table 3).

**Table 3:** User-friendliness of the Studied Devices.

Studied Device	NRS			
	Min	Max	Mean (SD)	*p* Value
Standard Gum Elastic Bougie	5	10	7.6 (1.5)	0.02
Flexible tip Bougie	3	8	5.8 (2.9)	

NRS—Numerical Rating Scale, SD—Standard Deviation.

## Data Availability

Data is contained within the article.
